# VRK1 (Y213H) homozygous mutant impairs Cajal bodies in a hereditary case of distal motor neuropathy

**DOI:** 10.1002/acn3.51050

**Published:** 2020-05-04

**Authors:** Ana T. Marcos, Elena Martín‐Doncel, Patricia Morejón‐García, Iñigo Marcos‐Alcalde, Paulino Gómez‐Puertas, María Segura‐Puimedon, Lluis Armengol, José M. Navarro‐Pando, Pedro A. Lazo

**Affiliations:** ^1^ Unidad de Genética Instituto para el Estudio de la Biología de la Reproducción Humana (INEBIR) Sevilla Spain; ^2^ Molecular Mechanisms of Cancer Program Instituto de Biología Molecular y Celular del Cáncer Consejo Superior de Investigaciones Científicas (CSIC) Universidad de Salamanca Salamanca Spain; ^3^ Instituto de Investigación Biomédica de Salamanca (IBSAL) Hospital Universitario de Salamanca Salamanca Spain; ^4^ Molecular Modelling Group Centro de Biología Molecular “Severo Ochoa” CSIC‐Universidad Autónoma de Madrid, Cantoblanco Madrid Spain; ^5^ School of Experimental Sciences Biosciences Research Institute Universidad Francisco de Vitoria Pozuelo de Alarcón, Madrid Spain; ^6^ Quantitative Genomic Medicine Laboratories, qGenomics Espluges de Llobregat Barcelona Spain; ^7^ Cátedra de Reproducción y Genética Humana Facultad de Ciencias de la Salud Universidad Europea del Atlántico Santander Spain; ^8^ Fundación Universitaria Iberoamericana (FUNIBER) Barcelona Spain

## Abstract

**Background:**

Distal motor neuropathies with a genetic origin have a heterogeneous clinical presentation with overlapping features affecting distal nerves and including spinal muscular atrophies and amyotrophic lateral sclerosis. This indicates that their genetic background is heterogeneous.

**Patient and methods:**

In this work, we have identified and characterized the genetic and molecular base of a patient with a distal sensorimotor neuropathy of unknown origin. For this study, we performed whole‐exome sequencing, molecular modelling, cloning and expression of mutant gene, and biochemical and cell biology analysis of the mutant protein.

**Results:**

A novel homozygous recessive mutation in the human *VRK1* gene, coding for a chromatin kinase, causing a substitution (c.637T > C; p.Tyr213His) in exon 8, was detected in a patient presenting since childhood a progressive distal sensorimotor neuropathy and spinal muscular atrophy syndrome, with normal intellectual development. Molecular modelling predicted this mutant VRK1 has altered the kinase activation loop by disrupting its interaction with the C‐terminal regulatory region. The p.Y213H mutant protein has a reduced kinase activity with different substrates, including histones H3 and H2AX, proteins involved in DNA damage responses, such as p53 and 53BP1, and coilin, the scaffold for Cajal bodies. The mutant VRK1(Y213H) protein is unable to rescue the formation of Cajal bodies assembled on coilin, in the absence of wild‐type VRK1.

**Conclusion:**

The VRK1(Y213H) mutant protein alters the activation loop, impairs the kinase activity of VRK1 causing a functional insufficiency that impairs the formation of Cajal bodies assembled on coilin, a protein that regulates SMN1 and Cajal body formation.

## Introduction

Hereditary neuropathies are characterized by involvement of motor, sensory, and/or autonomic nerve fibers,^1^ and are divided into three main categories: hereditary motor and sensory neuropathies (HMSN), also known as Charcot–Marie‐Tooth (CMT) disease, hereditary motor neuropathy, and hereditary sensory and autonomic neuropathy (HSAN).^2^ Distal neuropathies and spinal muscular atrophy (SMA) are progressive diseases affecting the lower motor neurons and characterized for a progressive muscle loss and weakness, and have overlapping symptoms.^3^ The most common forms of these diseases are associated with deletions or mutations in *CMT* genes, or in the exon of the *SMN1* gene that is not compensated by *SMN2* ^3^, ^4^ and is involved in RNA processing mediated by snRNP.^5^ However, there is heterogeneity in disease presentation, evolution and manifestations, which has led to the identification of novel genes implicated in the pathogenesis of these diseases.^3^ These include alterations in mechanisms that regulate RNA splicing and processing, or in the subcellular structures where these functions take place, such as Cajal bodies.

Cajal bodies (CBs) are assembled on coilin.^6^, ^7^ CBs organization and assembly are lost by either SMN1 depletion,^8^ or VRK1 depletion,^7^ and both form part of a common complex.^6^, ^7^ Coilin also forms complexes with splicing snRNP.^9^, ^10^, ^11^ Moreover, both SMN1^12^ and VRK1^13^ bind to chromatin. Coilin levels and its posttranslational modifications also affect RNA processing and splicing.^14^ In this context, VRK1 phosphorylates coilin in Ser184 and regulates its stability and assembly by protecting it from ubiquitin‐mediated proteasomal degradation in the cytosol, facilitating its nuclear accumulation,^7^ and also interacts with Heterogeneous nuclear ribonucleoprotein A1 (hnRNP A1) and regulates telomerase activity and telomere maintenance.^15^ CBs alterations can be alternative pathogenic routes leading to distal neuromotor syndromes, and in which multiple genes have been implicated.^16^

Several very rare recessive mutations in the human *VRK1* gene, either homozygous or compound heterozygous, have been detected in diseases affecting the motor neuron, which have a phenotypic heterogeneity in their clinical presentation.^17^, ^18^, ^19^, ^20^, ^21^, ^22^, ^23^, ^24^ These *VRK1* mutations are recessive, and all of them are very rare, some hereditary, and others de novo. Among the distal motor neuropathy phenotypes associated with human *VRK1* mutations are SMA,^17^, ^20^, ^23^, ^24^, ^25^ ALS,^19^, ^20^ and pontocerebellar hypoplasia.^17^

In this work, we have identified a novel homozygous recessive mutation in the human *VRK1* gene, and the mutant protein has altered the folding of its activation loop that prevents the activation of the kinase activity leading to a deficiency in the assembly of Cajal bodies.

## Patient, Materials, and Methods

### Clinical characteristics of the patient

The patient, son of consanguineous parents and currently 35 years, presented initial symptoms at 4 years with a progressive distal muscle weakness in legs and arms that became much more severe with time. The child has a foot deformity with pes cavus and bilateral foot drop, leading to unstable walk with distal amyotrophy of lower and upper members. Electromyogram, performed at 9 years, detected a significant slowdown of motor and sensory nerve conductance velocity. At 16 years required foot surgical correction to allow for adequate standing. The disease progressed with time needing walking stick, and currently is wheel chair bound. At 24 years there was a significant loss of muscle strength, unable to raise from sedestation without help, and at 34 years the patient cannot use hands for feeding or writing. Normal intellectual development and normal speech.

The parents are consanguineous first cousins, and the father has epilepsy. Both parents have pes cavus.

### Whole‐exome sequencing

For whole‐exome sequencing (WES), DNA was extracted from peripheral blood of the patient using the Maxwell 16 system. DNA quality was determined measuring optical density with a DeNovix DS‐11. Human exome was enriched using the MedExome SeqCap EZ assay (Roche‐Nimblegen, CA), and sequenced using a NextSeq 500 (Illumina, CA) equipment. An average 71x coverage depth was achieved along the target regions (exons and ± 75 intronic nucleotides lanking the exon–intron boundaries). Variants were identified by alignment of reads against the Human reference genome sequence (hg19), using BWA‐0.7–12, GATK‐3.5 procedures for the detection of single nucleotide variants and indels and exome depth for deletions and duplications. Annotation of the variants was performed using Annovar (2016Feb01) and filtering and prioritization was performed using public (1000 genomes, Genome Aggregation Database and Exome Variant Server) databases in order to identify candidate variants related to the clinical phenotype. Variants were classified following the American College of Medical Genetics and Genomics (ACMG) guidelines,^26^, ^27^ Polyphen‐2^28^ and VarElect^29^ algorithms were used to predict the effect of the variants. Sanger sequencing in the index case, parents, and siblings was performed to confirm the variant and to determine its inheritance pattern.

### Ethical compliance

The genomic study was performed for diagnosis in a case of unknown origin and was approved by the Institutional review board. Informed consent was obtained from each subject.

### Molecular modeling of structure

The 3D structure of the human Vaccinia‐Related Kinase 1 (VRK1) wild‐type protein was obtained from the Protein Data Bank (PDB id: http://www.rcsb.org/pdb/search/structidSearch.do?structureId=2LAV).^30^ The dynamic molecular modeling methods are described in Data S1.

### Cloning of altered VRK1 gene, plasmids, and mutagenesis

Human VRK1 was expressed from mammalian expression vector, pCEFL‐HA‐VRK1,^31^ and bacterial expression pGEX‐4T‐VRK1.^31^, ^32^, ^33^, ^34^ The Y213H mutation was introduced in these plasmids with the GeneArt Site‐Directed Mutagenesis System (Invitrogen‐ThermoFisher). The primers for human VRK1‐p.Y213H were: forward (5′‐ AGGAGTTCATAAAGAACACAAAGAAGACCCCAAAA‐3′), and reverse (5′‐TTTTGGGGTCTTCTTTGTGTTCTTTATGAACTCCT‐3′). VRK1 wild‐type and the p.Y213H variant proteins were expressed in *E.coli* from constructs made in plasmid pGEX4T‐GST‐VRK1 for bacterial expression,^32^ or in pCEFL‐HA‐VRK1 for expression in mammalian cells.^34^ The human Y213H mutation was introduced in murine *VRK1* (mVRK1) and cloned in pCEFL‐Myc‐mVRK1 to generate pCEFL‐Myc‐mVRK1(Y213H) plasmid. The primers used for murine VRK1‐Y213H forward (5′‐ TGGAGTTCATAAAGAGCACAAGGAAGATCCCAAA‐3′) and reverse (5′‐ TTTGGGATCTTCCTTGTACTCTTTATGAACTCCA‐3. Full methods are in Data S1.

### Kinase assays

Kinase assays were performed as previously described.^31^, ^34^, ^35^ Briefly, in vitro kinase assays with [^32^‐ P]‐γATP were performed with GST‐VRK1 wild‐type, or the p.Y213H variant.^7^, ^31^, ^36^ Kinase assays with the following substrates were previously published: histone H3,^13^, ^33^ H2AX,^13^ p53,^37^, ^38^ 53BP1,^39^ and coilin.^7^ Full methods are in Data S1.

### Cell lines, transfections, and protein analysis

All molecular and cellular methods have been reported before and are detailed in cellular and in Data S1.^34^ Antibodies are in Table S1.

## Results

### Genetic findings

**Figure 1:**
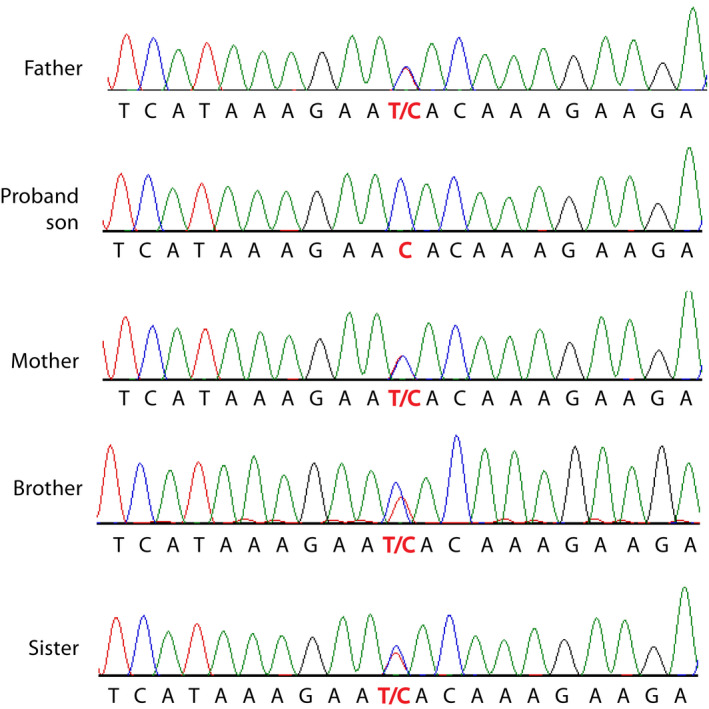
Identification of a novel mutation in the human *VRK1* gene coding for a nucleosomal kinase. Detection and confirmation of the *VRK1* mutation in the five members of the family.

Whole‐exome sequencing detected in the patient a homozygous mutation (NM_003384:exon8:c.T637C:p.Y213H) in exon 8 of the *VRK1* gene. This mutation was detected in heterozygosis in both consanguineous parents, and the two siblings of the patient (Fig. 1). Heterozygous carriers have no neurological pathology.

### Molecular modeling of VRK1(Y213H) variant protein and functional prediction

**Figure 2:**
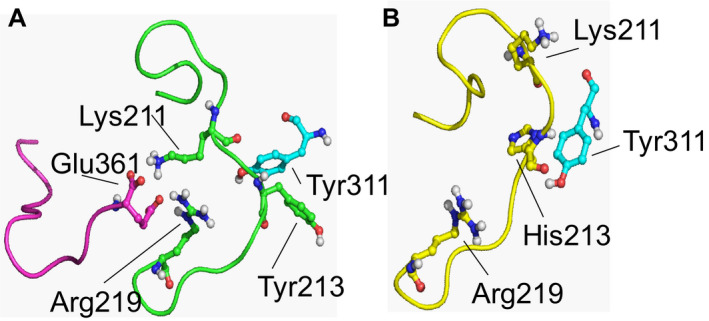
Structural modeling of the human VRK1‐Y213H variant protein. (A) Structure of the wild‐type VRK1 activation loop after 200 nsec of free molecular dynamics. Position of C‐terminal tail interacting with the activation loop is indicated (magenta). The activation loop is colored in green. Position of residues Tyr213, Tyr311 (blue, not in the activation loop), Lys211, Arg219 and Glu361 (grey, located in the C‐terminal tail) is indicated. (B) Structure of the activation loop of VRK1‐Y213H variant after 200 nsec of free molecular dynamics. Note the displacement of the loop compared to the wild‐type structure and the different position of the mutated residue (His213). No interaction was detected between the mutated activation loop and the C‐terminal tail of VRK1.

Polyphen‐2 analysis of this aminoacid substitution predicts the highest damaging score. VarElect analysis predicts a very high likelihood of causing a neuropathy and muscular atrophy.^29^ To study the effect of the VRK1(Y213H) mutant protein, the 3D structure of human VRK1 protein (2LAV) was obtained from the Protein Data Bank.^30^ This structure includes the position of the protein C‐terminal tail, as well as the structure of the VRK1 activation loop, where the Tyr213 residue is located (Fig. S1). Tyr213 forms a T‐shaped stacking complex with Tyr311, located in an alpha helix close to the activation loop (Fig. S1). This type of Tyr‐Tyr interactions has been shown as contributing to structural protein stability,^40^ as is the case of the VRK1 activation loop. The mutant residue VRK1(Y213H) is located in the activation loop of the kinase.^30^ To detect the structural consequences of the Y213H mutant, models for wild‐type (VRK1‐WT), and Y213H mutant (VRK1‐Y213H) proteins were subjected to 200 nsec of free molecular dynamic (MD) simulation. The activation loop of the VRK1‐WT structure did not experience noticeable variations during the molecular dynamics trajectory, remaining virtually unchanged after 200 nsec of MD (Fig. 2A). The only variation suffered by the activation loop was the stabilization of the C‐terminal end of the protein, through the formation of saline bridges between the negative amino acid Glu361 and the positive residues Lys211 and Arg219 of the loop (Fig. 2A). This interaction remained stable for more than 40% of the total simulation time. After 200 ns of molecular dynamics (MD), the 3D structure of the VRK1(Y213H) mutant activation loop displayed a different arrangement (Fig. 2B). His213 continued to interact with Tyr311 but in the form of a parallel‐displaced stacking, which confers a completely different shape to the activation loop. Because of this, the position of Lys211 suffered a large displacement. The behavior of the VRK1(Y213H) mutant differs of the VRK1‐WT structure during the MD trajectory. The mutant does not have a close interaction between the specific residues of the activation loop and the residues located in the regulatory C‐terminal tail of the protein, which are required for kinase activation,^30^, ^41^ and this structural change predicts a loss of kinase activity.

### The VRK1(Y213H) variant kinase is functionally deficient

**Figure 3:**
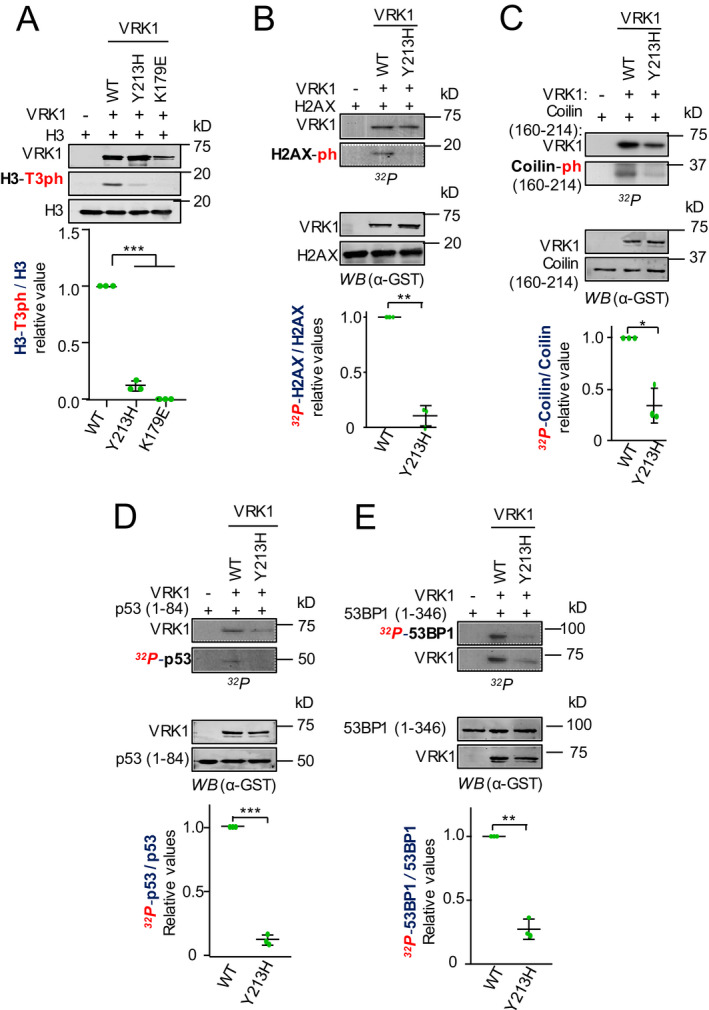
Kinase activity of the wild‐type VRK1 and mutant VRK1‐Y213H using different phosphorylation substrates. (A) Phosphorylation of histone H3 by wild‐type VRK1 (WT), VRK1‐T213H and kinase‐dead VRK1‐K179E in Thr3 detected with a phospho‐specific antibody. (B) Phosphorylation of histone H2AX in a radioactive kinase assay. (C) Phosphorylation of coilin in a radioactive kinase assay. (D) Phosphorylation of p53 in a radioactive kinase assay. (E) Phosphorylation of 53BP1 in a radioactive kinase assay. One representative gel is shown, and the three independent experiments are shown is Figure S2.

To determine the functional effect of the VRK1(Y213H) mutant, we performed in vitro kinase assays using several of the known VRK1 substrates. These included histones H3 and H2AX associated with chromatin remodeling,^13^ coilin required for Cajal body formation,^7^ p53,^42^ and 53BP1 ^39^ involved in different aspects of DNA damage responses.^43^ The mutant VRK1(Y213H) was compared with the wild‐type VRK1. In the kinase assays, the effect of the p.Y213H mutant was common to all substrates. There was a significant loss regarding the phosphorylation of histone H3, (Fig. 3A), histone H2AX (Fig. 3B), coilin (Fig. 3C), p53 (Fig. 3D), and 53BP1(Fig. 3E).

### Protein stability of the VRK1(Y213H) mutant

Several of the known VRK1 mutants associated with neurological phenotypes also have a reduced protein stability.^34^ Therefore, we tested the stability of p.Y213H compared to the normal VRK1 protein. Plasmids expressing both proteins were transfected in 293T cells, which were treated with cycloheximide, and the level of protein was determined at different time points. The p.Y213H mutant protein was slightly less stable that normal VRK1 (Fig. S3).

### Effect of the VRK1(Y213H) mutant on the formation of Cajal bodies

**Figure 4:**
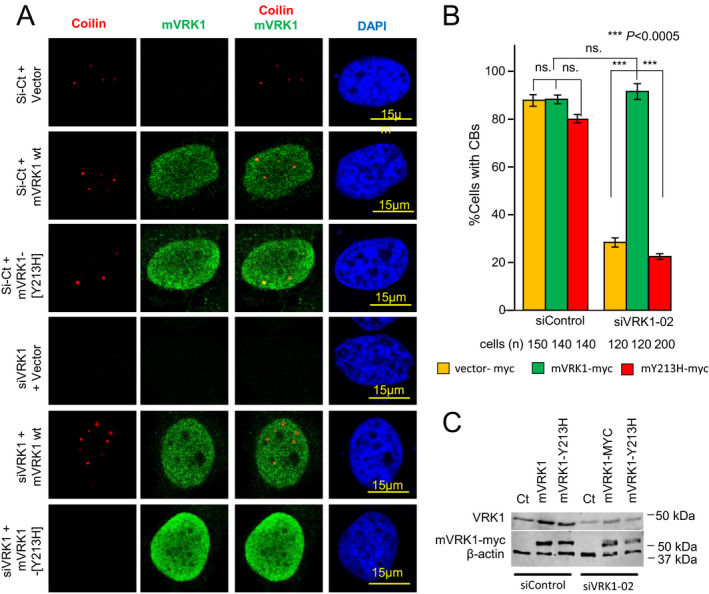
Rescue of Cajal body formation by wild‐type VRK1 and mutant VRK1‐Y213H. Endogenous human VRK1 was depleted with si‐VRK1‐02 and cells were transfected with either murine VRK1 wild‐type (muVRK1) or with the murine VRK1 Y213H variant (muVRK1‐Y213H). (A) Images of confocal microscopy showing the presence or absence of Cajal bodies assembled on coilin (red). (B) Quantification of the presence or absence of Cajal bodies in cells that after depletion of endogenous human VRK1 were transfected with either wild‐type of the Y213H mutant murine VRK1. (C) Immunoblot to show the depletion of endogenous human VRK1 and its replacement by either murine VRK1 or the murine Y213H VRK1 mutant.

Phosphorylation of coilin regulates the assembly and stability of Cajal bodies.^7^, ^44^ Therefore, we studied the possible effect of the Y213H mutant on the formation of Cajal bodies. For this purpose, we used Hela cells in which the endogenous human VRK1 was depleted by siRNA. These human VRK1 depleted cells were transfected with the murine *VRK1*, either wild‐type (mVRK1) or containing the Y213H mutant (mVRK1‐Y213H). In transfected cells depleted of human endogenous VRK1 there was a loss of Cajal bodies, and this effect was rescued in cells expressing the murine wild‐type VRK1, but not by the murine VRK1‐Y213H mutant protein (Fig. 4).

## Discussion

The heterogeneity of the neuromotor syndromes associated with VRK1 pathogenic variants suggest that their contribution is likely to be mediated by downstream direct targets. All pathogenic variants have in common two possible effects. Some have a reduction of the mutant protein stability, which leads to reduced protein levels. Others have a reduction of their kinase activity with respect to several of its known specific target proteins,^34^ some of which are already associated with neurological development and diseases, such as proteins regulating chromatin epigenetic changes, neural development or response to cellular stress and DNA damage.

In this report, we have shown that the VRK1(Y213H) mutant protein is unable to phosphorylate several of its substrates including histone H3, H2AX, p53, 53BP1, and coilin, all of them are pathogenically associated with hereditary neurological/neuromotor syndromes. Functionally, this impaired VRK1 kinase activity implicates a loss of functions, which have to be linked to distal motor neuropathies and SMA phenotype. The most likely intermediate step in the pathogenesis VRK1(Y213H) is a consequence of the inability of the Y213H mutant protein to phosphorylate and regulate coilin, which can alter its associated proteins and functions. Coilin is the scaffold protein of Cajal Bodies^45^ discovered more than one hundred years ago,^46^ which forms a complex with SMN1 in SMA.^6^ However, the function of Cajal bodies is not well understood, but it is a ribonucleoprotein complex that participates in RNA processing.^47^, ^48^ In a murine coilin knockout models there is a failure to recruit SMN1 to defective or absent CBs.^49^, ^50^ All human VRK1 mutant proteins, directly or indirectly fail to form CBs^7^, ^34^, ^51^ and mimic the coilin knockout. Furthermore, SMN1 protects against mutant SOD1 toxicity.^52^ Mutations in SOD1 disrupt the recruitment of SMN1 to Cajal bodies,^53^ and mutations in VCP (valosin) contribute to development of ALS in KO mice,^54^ all these proteins form complexes with VRK1 (unpublished).

Most of the known human VRK1 pathogenic variants are unable to form CBs,^34^ as it also occurs with the Y213H mutant. Moreover, in cells obtained from patients with compound R219L/W254L mutations, there are no CBs in motor neurons derived from human induced pluripotent stem cells.^51^ In a murine model, the human VRK1(R358X) mutant impairs cell cycle progression and migration of neuronal progenitors in early embryogenesis.^55^ Moreover, VRK1 partial knockdown in mice causes a motor dysfunction.^56^ Nuclear bodies, such as CBs, are regulated during embryogenesis,^57^ and their alteration is very likely to cause neurological disorders. In this context, VRK1 regulates the assembly and disassembly of CB mediated by a specific phosphorylation of coilin that controls its stability and the formation of complexes with other proteins.^7^ Thus, functionally deficient VRK1 mutations alter the physiological dynamics of CBs, and consequently of its associated proteins and functions. This has been shown to be the case for Y213H (this report) and for other mutants.^34^ The heterogeneity of the VRK1 mutations suggests that there might be differences in their specific protein interactions that lead to some heterogeneity among the associated neuromotor syndromes by altering the functionality of Cajal bodies.

Another target of VRK1, p53, regulates WRAP53 that is required for CB formation and maintenance of genome integrity,^58^, ^59^ but the activation of p53 by phosphorylation is impaired in the case of the VRK1(Y213H) mutant protein. CBs are associated with ribonucleoproteins complexes involved in RNA processing, but the consequences of their alteration is unknown.^47^, ^60^ In the CBs assembled on coilin there are other proteins such as SMN1, associated with SMA,^4^ VCP associated with amyotrophic lateral sclerosis (ALS),^61^ and ataxin 1 (ATXN1) associated with ataxia.^62^ Alterations in any of them cause a distal neuromotor phenotype.

All the VRK1 mutants are either homozygous or compound heterozygous. The heterogeneity of the neurological phenotypes associated with VRK1 pathogenic variants has some common features. All patients presented a functional alteration of motor neuron function, mainly as distal motor neuropathies, spinal muscular atrophy (SMA) or amyotrophic lateral sclerosis (ELA). However, they differed in their severity and age of presentation, although initial symptoms started during infancy in most cases and are progressive. There are also important differences in intellectual ability ranging from normal to very severe deficiency. Some additional neurological symptoms are associated. This heterogeneity is unlikely to be only the result of the specific VRK1 mutant, since molecularly they have a similar defective function,^34^ but rather by the contribution of additional variants in other genes, which differ among patients. However, when more than one case occurs within a family sharing the same mutant, the phenotype is similar among the affected family members.^17^

An alternative pathogenic mechanism implicating the contribution of VRK1 may be a consequence of its interaction with GARS,^41^ a gene whose mutations are also associated with distal neuropathies.^63^, ^64^, ^65^ However, the role of aminoacyl‐tRNA‐synthetases in these diseases is unknown, but may be a consequence of altering neurite formation^66^ and peripheral axons.^67^, ^68^

We conclude that *VRK1* mutations, associated with distal neuromotor syndromes of unknown origin, should be considered as a rare underlying pathogenic mechanism. Since defective CBs assembly and associated functions are common to a group of heterogeneous distal neuromotor syndromes, whose final neurological phenotype will be determined by the genetic background of the affected individual. Distal motor neuropathies are a consequence of a mutation in any of the different components of nuclear suborganelles, such as CBs, and which lead to this group of related neurological phenotypes.

## Conflict of Interest

The authors declare they have no competing conflict of interest.

## Author Contributions

ATM, MS‐P, and LA performed the genetic and sequencing study. EM‐D and PM‐G performed biochemical and cell biology studies, IM‐A and PG‐P performed molecular structural analysis, JMN‐P, and PAL planned the work and coordinated clinical and molecular studies. PAL wrote the final version of the manuscript that was revised and approved by all authors.

## Informed Consent

The parents and the affected patient provided written consent to perform the genetic study.

## Consent to Publish

Written informed consent for publication was obtained from the participants in the study.

## Supporting information


**Figure S1.** Structural modeling of VRK1 Y213H variant. (A) 3D structure of wild‐type VRK1 protein (PDB id: http://www.rcsb.org/pdb/search/structidSearch.do?structureId=2LAV). The activation loop is colored in green. Position of residues Tyr213, Tyr311 (blue, not in the activation loop), Lys211, Arg219 and Glu361 (grey, located in the C‐terminal tail) are indicated.

**Figure S2.** Phosphorylation of several substrates by VRK1 and VRK1‐Y213H in triplicate. (A) Histone H3. (B) Histone H2AX. (C) Coilin. (D) TP53. (E) 53BP1.

**Figure S3.** Protein stability of the wild‐type VRK1 and mutant VRK1‐Y213H proteins.

**Table S1.** List of primary and secondary antibodies used in this work.

**Data S1.** This refers to databases correctly placed before in the text.

## Data Availability

The *VRK1* mutation is available in ClinVar with the variation ID 812546. https://www.ncbi.nlm.nih.gov/clinvar/variation/812546/ WES datasets are available at https://www.ncbi.nlm.nih.gov/sra/PRJNA606693.
