# Supplementary material for: VRK1 (Y213H) homozygous mutant impairs Cajal bodies in a hereditary case of distal motor neuropathy
Source: Ann Clin Transl Neurol. 2020 May 4;7(5):808–18. doi: 10.1002/acn3.51050 (PMC7261760; doi:10.1002/acn3.51050)
Supplement: Supplementary file 1 — Figure S1. Structural modeling of VRK1 Y213H variant. (A) 3D structure of wild‐type VRK1 protein (PDB id: http://www.rcsb.org/pdb/search/structidSearch.do?structureId=2LAV). The activation loop is colored in green. Position of residues Tyr213, Tyr311 (blue, not in the activation loop), Lys211, Arg219 and Glu361 (grey, located in the C‐terminal tail) are indicated. [file ACN3-7-808-s001.pdf]

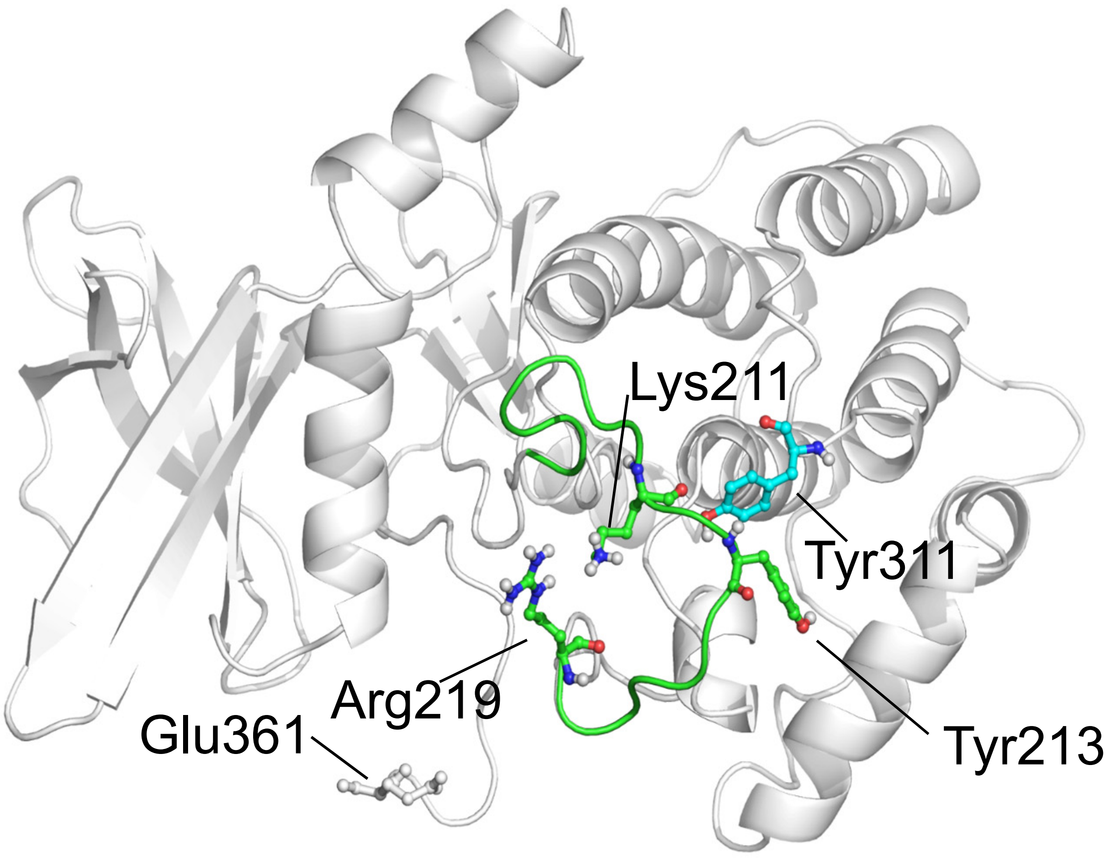

Supplementary Figure S1. Structural modeling of VRK1 Y213H variant. (A) 3D structure of wild-type VRK1 protein (PDB id: 2LAV). The activation loop is colored in green. Position of residues Tyr213, Tyr311 (blue, not in the activation loop), Lys211, Arg219 and Glu361 (grey, located in the C-terminal tail) is indicated.
