# Supplementary material for: VRK1 (Y213H) homozygous mutant impairs Cajal bodies in a hereditary case of distal motor neuropathy
Source: Ann Clin Transl Neurol. 2020 May 4;7(5):808–18. doi: 10.1002/acn3.51050 (PMC7261760; doi:10.1002/acn3.51050)
Supplement: Supplementary file 2 — Figure S2. Phosphorylation of several substrates by VRK1 and VRK1‐Y213H in triplicate. (A) Histone H3. (B) Histone H2AX. (C) Coilin. (D) TP53. (E) 53BP1. [file ACN3-7-808-s002.pdf]

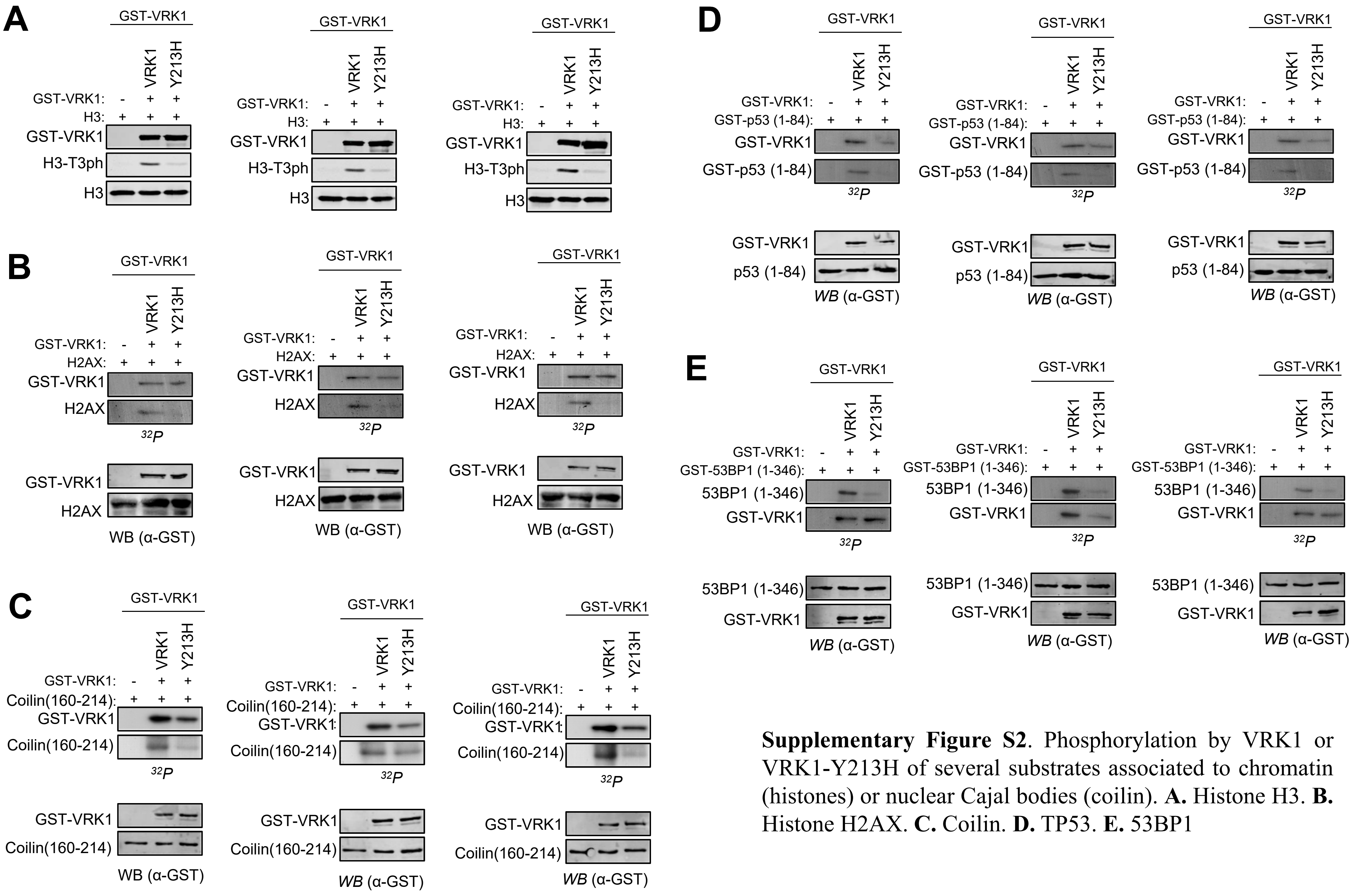

Supplementary Figure S2. Phosphorylation by VRK1 or VRK1-Y213H of several substrates associated to chromatin (histones) or nuclear Cajal bodies (coilin). **A.** Histone H3. **B.** Histone H2AX. **C.** Coilin. **D.** TP53. **E.** 53BP1
