# Supplementary material for: VRK1 (Y213H) homozygous mutant impairs Cajal bodies in a hereditary case of distal motor neuropathy
Source: Ann Clin Transl Neurol. 2020 May 4;7(5):808–18. doi: 10.1002/acn3.51050 (PMC7261760; doi:10.1002/acn3.51050)
Supplement: Supplementary file 3 — Figure S3. Protein stability of the wild‐type VRK1 and mutant VRK1‐Y213H proteins. [file ACN3-7-808-s003.pdf]

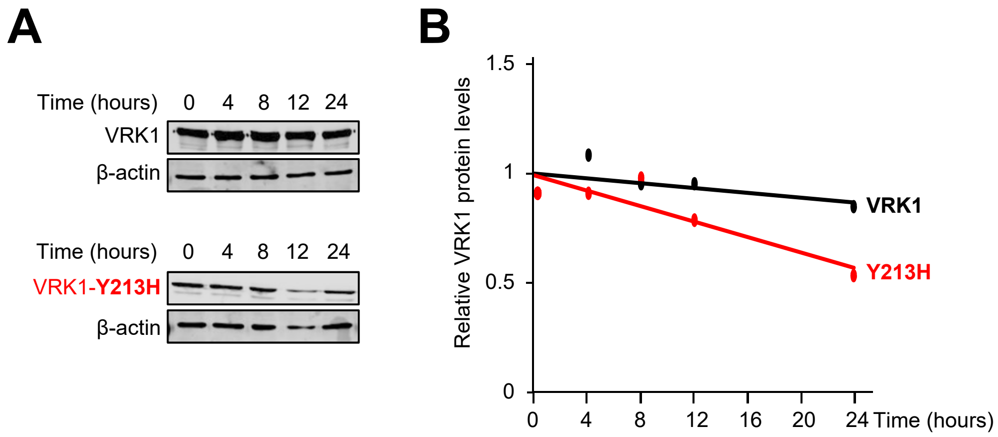

Supplementary Figure S3. Protein stability of the wild-type VRK1 and mutant VRK1-Y213H proteins. Plamids expressing human VRK1 or VRK1-Y213H tagged with the HA-epitope were cloned in plasmid pCEFL-HA vector and transfected in HEK293T cells. Cycloheximide was added to the culture to block gene transcription. The level of VRK1 proteins after cycloheximide addition were determined in westen blots. **A.** The experiment was performed in triplicate and a representative gel is shown. **B.** The relative level of each protein at different times was quantitated and shown in the graph (B).
