# Supplementary material for: VRK1 (Y213H) homozygous mutant impairs Cajal bodies in a hereditary case of distal motor neuropathy
Source: Ann Clin Transl Neurol. 2020 May 4;7(5):808–18. doi: 10.1002/acn3.51050 (PMC7261760; doi:10.1002/acn3.51050)
Supplement: Supplementary file 4 — Table S1. List of primary and secondary antibodies used in this work. [file ACN3-7-808-s004.docx]

**Supplementary Table S1**. List of primary and secondary antibodies used in this work.

| **Antibody** | **Type** | **Dilution WB** | **Clone and/or reference** | **Supplier** |
| --- | --- | --- | --- | --- |
| GST-Tag | Mouse monoclonal | 1:1000 | B14/sc-138 | Santa Cruz |
| HA-Tag | Mouse monoclonal | 1:1000 | F7/sc-7392 | Santa Cruz |
| Histone H3 | Rabbit polyclonal | 1:1000 | 9175 | Cell Signaling |
| Phospho-histone H3 (Thr3ph) | Rabbit polyclonal | 1:1000 | 05-746R | Merck-Millipore |
| coilin | Mouse monoclonal | 1:200 | [sc-56298](https://www.scbt.com/es/p/coilin-antibody-pdelta?requestFrom=search) | Santa Cruz |
| β-actin | Mouse monoclonal | 1:1000 | AC15/A5441 | Sigma-Aldrich |
| Goat Anti-Mouse IgG, DyLight 680 | Goat | 1:10000 | 35518 | Thermo Scientific |
| Goat Anti-Rabbit IgG, DyLight 800 | Goat | 1:10000 | 35571 | Thermo Scientific |
| Anti-Mouse IgG-HRP | Sheep | 1:10000 | NA931V | Amersham Biosciences; |
