# Supplementary material for: VRK1 (Y213H) homozygous mutant impairs Cajal bodies in a hereditary case of distal motor neuropathy
Source: Ann Clin Transl Neurol. 2020 May 4;7(5):808–18. doi: 10.1002/acn3.51050 (PMC7261760; doi:10.1002/acn3.51050)
Supplement: Supplementary file 5 — Data S1. This refers to databases correctly placed before in the text. [file ACN3-7-808-s005.pdf]

Supplementary material and methods

Structural modeling of VRK1-Y213H variant

The 3D structure of the human Vaccinia-Related Kinase 1 (VRK1) wild-type protein was obtained from the Protein Data Bank (PDB id: 2LAV)[1]. The first conformer (of the 20 NMR conformers included in the PDB file) was selected for further processing. Model for VRK1-Y213H variant was generated using the wild-type structure as template. Models were built using the SWISS-MODEL server (http://swissmodel.expasy.org) and their structural quality were within the range of those accepted for homology-based structure (Anolea/Gromos/QMEAN4) [2].

Molecular Dynamics simulation

Prior to molecular dynamics (MD) procedures, 3D structures were energy minimized using the GROMOS 43B1 force field implemented in DeepView (http://spdbv.vital-it.ch/), using 500 steps of steepest descent minimization followed by 500 steps of conjugate-gradient minimization.

Wild-type VRK1 structure and VRK1-Y213H model were subjected to 200 ns of MD simulation using the AMBER18 molecular dynamics package (http://ambermd.org/; University of California-San Francisco, CA). The 3D structures were solvated with a periodic octahedral pre-equilibrated solvent box using the LEaP module of AMBER, with 12 Å as the shortest distance between any atom in the protein subdomain and the periodic box boundaries. Free MD simulation were performed essentially as previously described [3], using the PMEMD program of AMBER18 and the ff14SB force field (http://ambermd.org/). The SHAKE algorithm was used, allowing a time step of 2 fs. Systems were initially relaxed over 15,000 steps of energy minimization with a cut-off of 12 Å. Simulations were then started with a 20 ps heating phase, raising the temperature from 0 to 300 K in 10 temperature change steps, after each of which velocities were reassigned. During minimization and heating, the Cα trace dihedrals were restrained with a force constant of 500 kcal mol^{-1} rad^{-2} and gradually released in an equilibration phase in which the force constant was progressively reduced to 0 over 200 ps. After the equilibration phase, 200 ns of unrestricted MD simulation were obtained. MD trajectories were analyzed using VMD software [4]. The trajectories were continuously monitored by the measurement of root-mean square deviation (RMSD).

Figures were generated using the Pymol Molecular Graphics System (https://pymol.org/; Schrödinger, LLC, Portland, OR).

Plasmids and mutagenesis

Human VRK1 was expressed from mammalian expression vector, pCEFL-HA-VRK1 [5], and bacterial expression pGEX-4T-VRK1 [5-8]. The following primers were used to generate the Y213H mutations in human and murine VRK1. Mutations in VRK1 were performed using the GeneArt Site-Directed Mutagenesis System (Invitrogen-ThermoFisher) with the following primers for human VR K1-Y213H forward (5'-AGGAGTTCATAAAGAACACAAAGAAGACCCCAAAA-3') and reverse (5'-TTTTGGGGTCTTCTTTGTGTTCTTTATGAACTCCT-3'); and for murine VRK1-Y213H forward (5'- TGGAGTTCATAAAGAGCACAAGGAAGATCCCAAA-3') and reverse (5'- TTTGGGATCTTCCTTGTACTCTTTATGAACTCCA-3). Sanger sequencing was used to confirm all variants generated.

Human VRK1 wild type and the VRK1-Y213H variant were expressed from constructs pGEX4T-GST-VRK1 plasmid expressed in *E.coli* BL21 strain competent cells. The following plasmids were used to express the substrates: pGST4T-53BP1 (1-346) [9, 10]; GST-p53(1-85)[11] [6, 12], and pGEX4T-GST-Coilin(160-214) [13]. All plasmids were expressed in BL21 *E.coli* to purify the fusion protein used as substrate in kinase assays as previously reported [8, 13]. Human H3 is a purified recombinant protein (Merck-Millipore).

Kinase assays

The kinase assays were performed as previously described [5, 8, 14]. Briefly, In vitro kinase assays with [^{32}- P]-γATP were performed with GST-VRK1 wild-type and variants [5, 13, 15]. Assays with the following substrates were previously published: p53 [12, 16], histone H3 [7, 17], 53BP1 [10], and GST-coilin [13].

The Serine-Threonine kinase activity of VRK1 was analysed by performing in vitro kinase assays using 2 µg of GST-VRK1 and variant recombinant proteins that were purified from BL21 cells. The following proteins (2 µg) were used as specific substrates, GST-53bp1(1-346) [10] , GST-Coilin(160-214) [13], GST-p53(1-84) [6, 18, 19], and human recombinant histones H3 [7, 17]. To perform the in vitro kinase assay [19], it was used a specific buffer (20mM Tris-HCl pH 7.5, 5mM $MgCl_2$, 0.5mM DTT

and 150mM KCl), 5µM ATP and 5µCi (0.1µM) radiolabelled [ɣ-^{32}P]ATP in a final volume of 40µl during 45 min at 30ºC [8]. H3T3ph was detected with a rabbit polyclonal antibody (Upstate-Millipore) [15, 17]. In radioactive assays, film exposure was in the lineal response range for all assays.

Electrophoresis, antibodies and immunoblots

Proteins were separated in SDS-PAGE gels in running buffer (25mM Tris-HCl, ph 8.0, 200mM glycine, 1.7mM SDS), and transferred to a PVDF membrane (Immobilon-FL, Millipore) in buffer (25mM Tris-HCl, pH 8.0, 19.2 mM glycine, 15% methanol) as previously described [13, 15, 20, 21]. The primary and secondary antibodies are listed in Supplementary Table S1. The secondary antibodies were incubated for an hour and the fluorescence was detected with LI-COR Odyssey Infrared Imaging System or with ECL Western Blotting Detection Reagent (Sigma-Aldrich) were used if the secondary antibodies were conjugated with peroxidase.

Cell lines, transfection and cell lysate

For the study of Cajal bodies formation, the validated HeLa (ATCC-CCL2) cell line was grown in Dulbecco's Modified Eagle's Medium (DMEM) (Sigma-Aldrich) and transfected using Lipofectin [13, 15, 20]. Cell extracts were prepared by using a mild lysis buffer (50mM Tris-HCl, pH 8.0, 150mM NaCl, 1% Triton X-100 and 1mM EDTA) supplemented with protease inhibitors (1mM PMSF, 10 µg/mL aprotinin and 10 µg/mL leupeptin) and phosphatase inhibitors (1mM sodium orthovanadate, 1mM NaF) and incubated for 20 minutes [8, 13, 15].

Statistical analysis

Statistical analysis were performed using the IBM SPSS 28 statistics package. All assays were performed in the lineal response range and in identical conditions for all substrates [8]. Individual quantitative experiments were repeated three times, and statistical significance was analyzed using two-tailed T-test with Welch' correction [22].

Reagents

Recombinant human histones H3 and H2AX (Millipore, Merck), Cycloheximide (Sigma-Aldrich). All other chemical were from Sigma-Merck (Darmstadt, Germany).

Tissue culture media and reagents were from GIBCO-ThermoFisher Scientific (Waltham, MA).

REFERENCES

1. Shin, J., Chakraborty, G., Bharatham, N., Kang, C., Tochio, N., Koshiba, S., Kigawa, T., Kim, W., Kim, K. T. & Yoon, H. S. (2011), *J Biol Chem.* **286**, 22131-8.
2. Benkert, P., Biasini, M. & Schwede, T. (2011), *Bioinformatics.* **27**, 343-50.
3. Marcos-Alcalde, I., Mendieta-Moreno, J. I., Puisac, B., Gil-Rodriguez, M. C., Hernandez-Marcos, M., Soler-Polo, D., Ramos, F. J., Ortega, J., Pie, J., Mendieta, J. & Gomez-Puertas, P. (2017), *Sci Rep.* **7**, 3266.
4. Humphrey, W., Dalke, A. & Schulten, K. (1996), *J Mol Graph.* **14**, 33-8, 27-8.
5. Vazquez-Cedeira, M., Barcia-Sanjurjo, I., Sanz-Garcia, M., Barcia, R. & Lazo, P. A. (2011), *PLoS ONE.* **6**, e23235.
6. Lopez-Borges, S. & Lazo, P. A. (2000), *Oncogene.* **19**, 3656-64.
7. Moura, D. S., Campillo-Marcos, I., Vazquez-Cedeira, M. & Lazo, P. A. (2018), *Cell Mol Life Sci.* **76**, 2591-2611.
8. Martin-Doncel, E., Rojas, A. M., Cantarero, L. & Lazo, P. A. (2019), *Sci Rep.* **9**, 13381.
9. Rappold, I., Iwabuchi, K., Date, T. & Chen, J. (2001), *J Cell Biol.* **153**, 613-20.
10. Sanz-Garcia, M., Monsalve, D. M., Sevilla, A. & Lazo, P. A. (2012), *J Biol Chem.* **287**, 23757-23768.
11. Milne, D. M., Campbell, D. G., Caudwell, F. B. & Meek, D. W. (1994), *J Biol Chem.* **269**, 9253-9260.
12. Lopez-Sanchez, I., Valbuena, A., Vazquez-Cedeira, M., Khadake, J., Sanz-Garcia, M., Carrillo-Jimenez, A. & Lazo, P. A. (2014), *FEBS Lett.* **588**, 692-700.
13. Cantarero, L., Sanz-Garcia, M., Vinograd-Byk, H., Renbaum, P., Levy-Lahad, E. & Lazo, P. A. (2015), *Sci Rep.* **5**, 10543.
14. Barcia-Sanjurjo, I., Vazquez-Cedeira, M., Barcia, R. & Lazo, P. A. (2013), *J Biol Inorg Chem.* **18**, 473-82.
15. Moura, D. S., Fernandez, I. F., Marin-Royo, G., Lopez-Sanchez, I., Martin-Doncel, E., Vega, F. M. & Lazo, P. A. (2016), *Sci Rep.* **6**, 28532.
16. Santos, C. R., Vega, F. M., Blanco, S., Barcia, R. & Lazo, P. A. (2004), *Virology.* **328**, 254-65.
17. Salzano, M., Sanz-Garcia, M., Monsalve, D. M., Moura, D. S. & Lazo, P. A. (2015), *Epigenetics.* **10**, 373-83.
18. Milne, D. M., Palmer, R. H., Campbell, D. G. & Meek, D. W. (1992), *Oncogene.* **7**, 1361-1369.
19. Barcia, R., Lopez-Borges, S., Vega, F. M. & Lazo, P. A. (2002), *Arch Biochem Biophys.* **399**, 1-5.
20. Monsalve, D. M., Campillo-Marcos, I., Salzano, M., Sanz-Garcia, M., Cantarero, L. & Lazo, P. A. (2016), *Biochim Biophys Acta Molecular Cell Research.* **1863**, 760-9.
21. Salzano, M., Vazquez-Cedeira, M., Sanz-Garcia, M., Valbuena, A., Blanco, S., Fernandez, I. F. & Lazo, P. A. (2014), *Oncotarget.* **5**, 1770-1778.
22. Bremer, M. & Doerge, R. M. (2009) *Statistics at the bench: a step-by step handbook for biologists*, Cold Spring Harbor Laboratory Press, New York.
