# Supplementary material for: VRK1 (Y213H) homozygous mutant impairs Cajal bodies in a hereditary case of distal motor neuropathy
Source: Ann Clin Transl Neurol. 2020 May 4;7(5):808–18. doi: 10.1002/acn3.51050 (PMC7261760; doi:10.1002/acn3.51050)
Supplement: Supplementary file 6 [file ACN3-7-808-s006.docx]

This refers to databases correctly placed before in the text.
